# Graphomotor performance as a quantitative marker of Alzheimer’s disease: a tablet-based case–control ROC study

**DOI:** 10.3389/fnins.2026.1900444

**Published:** 2026-08-04

**Authors:** Edward Jacek Gorzelańczyk, Ewa Laskowska, Katarzyna Pasgreta, Piotr Kamiński, Aleksandra Kawala-Sterniuk, Piotr Walecki

**Affiliations:** 1 Institute of Philosophy, Kazimierz Wielki University in Bydgoszcz, Bydgoszcz, Poland; 2 Faculty of Mathematics and Computer Science, Computational Psychiatry Team, Adam Mickiewicz University in Poznań, Poznań, Poland; 3 Medically Assisted Recovery Association “MAR”, Bydgoszcz, Poland; 4 Oskar Bielawski Greater Poland Neuropsychiatric Center in Kościan, Kościan, Poland; 5 Collegium Medicum, Nicolaus Copernicus University in Toruń, Bydgoszcz, Poland; 6 Department of Cardiology, Faculty of Medicine, Collegium Medicum, Nicolaus Copernicus University, Bydgoszcz, Poland; 7 Clinic of Psychiatry University Hospital No. 1, Antoni Jurasz Memorial, Collegium Medicum, Nicolaus Copernicus University, Bydgoszcz, Poland; 8 Department of Medical Biology and Biochemistry, Faculty of Medicine, Nicolaus Copernicus University in Toruń, Collegium Medicum in Bydgoszcz, Bydgoszcz, Poland; 9 Department of Nature Conservation, Institute of Biological Sciences, University of Zielona Góra, Zielona Góra, Poland; 10 Department of Artificial Intelligence, Wrocław University of Science and Technology, Wrocław, Poland; 11 Department of Bioinformatics and Telemedicine, Faculty of Medicine, Jagiellonian University Medical College, Krakow, Poland

**Keywords:** Alzheimer’s disease, digital biomarkers, graphomotor performance, handwriting, motor control, executive function, tablet-based assessment, trail making test

## Abstract

**Background:**

From the clinical and neurobiological perspective of cortico subcortical loop concepts, Alzheimer’s disease (AD) is associated with progressive deterioration of cognitive, emotional, and motor functions, which can be assessed subclinically using digital motion tracking technology in an electromagnetic digitizing tablet technology. Graphomotor analysis provides a source of sensitive digital biomarkers with potential applications in the early diagnosis and monitoring of AD.

**Methods:**

A case–control study was conducted including 72 patients with clinically diagnosed Alzheimer’s disease (AD) and 44 cognitively healthy controls. Participants performed three graphomotor tasks (signature, free drawing, and the Trail Making Test A/B) using a digitizing tablet. Temporal, kinematic, and pressure-related parameters were recorded, including execution time, path length, mean and maximum velocity, pressure variability on the surface, and total harmonic distortion indices. Temporal measures reflected the duration and timing structure of task execution, kinematic measures described movement speed and variability, and pressure-related measures quantified the force applied by the pen and its stability during graphomotor performance. Statistical analysis was performed using the Mann–Whitney *U* test, and diagnostic accuracy was evaluated with receiver operating characteristic (ROC) analyses.

**Findings:**

Patients with Alzheimer’s disease exhibited significantly longer execution times, reduced mean and maximum velocities, and greater variability of velocity and pressure compared with healthy controls. In the signature task, kinematic measures showed the strongest discriminatory power, whereas in the drawing task, pressure variability emerged as the most sensitive parameter. In the Trail Making Test A, patients with AD performed significantly worse than controls across temporal, kinematic, and pressure domains, and the majority were unable to complete part B. ROC analyses demonstrated that execution time in TMT A (AUC 0.79; 95% CI 0.71–0.86), maximum velocity in TMT B (0.77; 0.68–0.85), and pressure variability (0.78; 0.69–0.85) achieved the highest diagnostic discriminatory value.

**Interpretation:**

Digital analysis of biomechanical signals from graphomotor tasks indicates significantly reduced motor control and diminished visuospatial–executive functioning in individuals with Alzheimer’s disease (AD) compared with healthy controls. Patients with AD demonstrated longer task execution times, decreased movement velocity, and greater variability of pressure relative to the control group. These findings suggest that tablet-derived graphomotor parameters may serve as candidate digital markers of motor and executive dysfunction in Alzheimer’s disease. Further external validation and longitudinal studies are needed before their use as diagnostic biomarkers can be recommended.

## Introduction

Alzheimer’s disease (AD) represents the leading cause of dementia worldwide, with more than 55 million people currently affected and projections exceeding 150 million by 2050 (Alzheimer’s Disease International, 2022; GBD 2019 Dementia Forecasting Collaborators, 2022). Alzheimer’s disease, beyond its clinical dimension, raises profound questions about the nature of personhood and identity. The erosion of memory undermines the biographical continuity of the individual and challenges our understanding of dignity, which cannot be reduced to cognitive capacity alone. The ethical dimension of the disease is revealed in how communities respond to human fragility and dependence (Götzelmann et al., 2021).

Alzheimer’s disease imposes an immense and growing economic burden worldwide. The global societal costs of dementia were estimated at over 800 billion USD in 2015 and surpassed 1 trillion USD by 2018, with projections exceeding 2 trillion USD by 2030 (Alzheimer’s Disease International, 2022). Recent analyses confirm that informal care accounts for more than half of these costs, highlighting the disproportionate impact on families and caregivers (Cavaco et al., 2025). Biomarkers are now recognized as central to the early and differential diagnosis of Alzheimer’s disease (Dubois et al., 2023). The disorder has been reframed as a clinical–biological construct requiring integration of clinical features with fluid and imaging markers (Dubois et al., 2024). In parallel, blood-based biomarkers are emerging as scalable and translational tools with potential for routine clinical application (Hampel et al., 2018).

Despite advances in neuroimaging and fluid biomarkers, clinical diagnosis still relies heavily on neuropsychological testing, which often lacks sensitivity in prodromal stages and is confounded by demographic and practice effects (Jack et al., 2018). This diagnostic gap underscores the urgent need for objective, scalable, and ecologically valid biomarkers capable of capturing subtle dysfunction before overt cognitive decline emerges (Dubois et al., 2023). It has been demonstrated that mobile and wearable devices can provide valuable behavioral and physiological indicators that complement traditional diagnostic methods (Kourtis et al., 2019). The development of digital health technologies in the field of Alzheimer’s disease and related dementias is accelerating, with interdisciplinary collaboration facilitating their translation into clinical practice (Lott et al., 2024). Increasing attention is also being directed toward the identification of digital biomarkers that support early diagnosis and disease monitoring (Gramkow et al., 2025).

Digital biomarkers have gained prominence as a complementary approach, offering high-resolution, continuous measures of behavior and physiology through tablets, sensors, and wearable devices (Kourtis et al., 2019). Among these, graphomotor performance, captured during handwriting, drawing, or trail-making tasks, provides a unique window into the integrity of cortico-subcortical loops that integrate motor, cognitive, and visuospatial processes. Unlike conventional scoring, digital decomposition of pen-based tasks yields temporal, kinematic, and pressure-related parameters that can reveal early dysfunction invisible to standard assessments (Planton et al., 2013; Zham et al., 2017). The neurobiological rationale for graphomotor biomarkers is grounded in the functional architecture of cortico-striato-thalamo-cortical circuits, which connect prefrontal, parietal, and temporal cortical regions with the basal ganglia and thalamus, supporting executive control, visuospatial integration, and fine motor regulation (Alexander et al., 1986).

Neuropathological staging demonstrates that AD pathology progressively disrupts these loops, particularly fronto-parietal and temporo-hippocampal connections, leading to characteristic slowing, irregularity, and impaired motor precision (Braak and Braak, 1991; Haber, 2016). Functional imaging studies further confirm that reduced connectivity within these circuits correlates with both cognitive decline and motor slowing in AD (Sala et al., 2017). The striatum central structure of cortico-subcortical loops is considered to play a fundamental role in the pathophysiology of Alzheimer’s disease, with structural, metabolic, and vascular alterations contributing to cognitive, emotional and motor dysfunction (de Jong et al., 2008; Leh et al., 2007). Neuroimaging studies have demonstrated reduced volumes and atrophy of striatal structures, including the caudate nucleus and putamen, in patients with Alzheimer’s disease (de Jong et al., 2008; Madsen et al., 2010). Functional and metabolic changes within these circuits have also been reported, linking striatal dysfunction to cognitive decline and motor slowing (Leh et al., 2016). Diffusion tensor imaging studies have additionally identified compensatory white-matter changes within basal ganglia pathways in Alzheimer’s disease (Wurst et al., 2023). More recent experimental models indicate that vascular degeneration and fibrinogen extravasation in the striatum correlate with motor impairments, further underscoring its role in disease progression (Berk-Rauch et al., 2023).

Graphomotor tasks are ideally suited to probe these networks. Writing and drawing require the dynamic coordination of cortical planning regions, basal ganglia for motor sequencing, thalamic relay for sensorimotor integration, and cerebellar contributions to timing and smoothness. Alterations in execution time, velocity, and pressure variability observed in AD patients reflect the breakdown of these distributed loops, providing quantifiable markers of disease-related dysfunction (Werner et al., 2006). Importantly, such measures are non-invasive, inexpensive, and amenable to longitudinal monitoring, aligning with the translational goals of early detection and disease tracking. It has been indicated that in Alzheimer’s disease, motor disturbances may emerge either concurrently with or prior to the onset of cognitive impairments. The analysis of motor disturbances during gait, motor coordination, or graphomotor performance highlights their potential as indicators of disease progression (Andrade-Guerrero et al., 2024; Buchman and Bennett, 2011; Mirelman et al., 2014; Tufekcioglu et al., 2021).

Recent work has already shown that digital handwriting and drawing features can support the detection of cognitive decline in neurodegenerative disease. Automated analysis of drawing processes has demonstrated that features such as drawing speed, pen posture, pressure, pauses, and stroke dynamics can differentiate cognitively normal individuals from patients with MCI or dementia and can also relate to MMSE scores and medial temporal lobe atrophy (Yamada et al., 2022). Similarly, studies of handwriting and drawing kinematics have shown that pressure and movement features differ between healthy controls, MCI, and Alzheimer’s disease groups (Garre-Olmo et al., 2017). Digital versions of the Trail Making Test have also been developed to move beyond conventional completion-time scoring; for example, simultaneous analysis of hand and eye trajectories can decompose TMT performance into process-level markers of working memory and inhibitory control (Linari et al., 2022; Juantorena et al., 2024; Toro-Serey et al., 2024). More recent multimodal approaches, have extended this framework by combining drawing trajectory with facial-expression and hand-motion features during drawing-based MCI screening (Fan et al., 2025).

In this study, we extend the investigation of graphomotor dysfunction in AD by applying a comprehensive digital assessment protocol. By systematically analyzing temporal, kinematic, and pressure-related parameters across signature, drawing, and trail-making tasks, we aim to identify which features best discriminate AD patients from cognitively healthy controls. We further evaluate their diagnostic performance using receiver operating characteristic (ROC) analysis, testing the hypothesis that specific graphomotor markers can serve as potential non-invasive digital biomarkers of Alzheimer’s disease. Temporal parameters refer to measures describing the duration and timing structure of task performance, including total execution time and interruptions in pen contact. Kinematic parameters describe the dynamics of pen movement, including path length, mean velocity, maximum instantaneous velocity, and variability of instantaneous velocity. Pressure-related parameters quantify the relative force exerted by the pen on the tablet surface, including mean pressure, maximum pressure, and pressure variability. Together, these measures provide complementary information on psychomotor speed, visuomotor coordination, motor fluency, and fine force regulation. The selected graphomotor parameters were chosen because they reflect components of motor-cognitive integration that are vulnerable in Alzheimer’s disease. Execution time and movement velocity provide indices of psychomotor slowing, a common manifestation of impaired cortico-subcortical processing. Variability of velocity and trajectory reflects reduced motor fluency and impaired online movement regulation, whereas pressure variability captures instability of fine force control. Since handwriting, drawing, and trail-making require the coordinated engagement of visuospatial processing, executive planning, working memory, and motor sequencing, abnormalities in these parameters may reflect disease-related disruption of distributed motor-cognitive networks rather than isolated peripheral motor impairment.

In this context, the novelty of the present study does not lie in the use of graphomotor assessment itself, but in the direct comparison of three clinically established graphomotor tasks, signature production, structured drawing, and digital TMT A/B, within a single Alzheimer’s disease cohort using one standardized tablet-based acquisition system. Unlike multimodal frameworks (Fan et al., 2025), our protocol focuses exclusively on graphomotor signals, making it simpler, more reproducible, and easier to implement in routine neuropsychological practice. At the same time, we acknowledge that the absence of multimodal behavioural features, external validation, and multivariable machine-learning models represents an important limitation of the present study.

## Methods

### Study design and participants

This study compared patients with a confirmed diagnosis of Alzheimer’s disease to cognitively healthy controls, with the objective of evaluating divergences in mental functioning metrics and clinical profiles. The clinical group comprised 72 individuals who met the ICD-10 diagnostic criteria for probable Alzheimer’s disease, established through comprehensive psychiatric, neurological, and neuropsychological assessment aimed at enhancing diagnostic accuracy. All participants underwent routine neuroimaging and a standard laboratory panel to exclude secondary causes of cognitive decline. Patients were consecutively recruited from a memory disorders outpatient clinic and a clinical psychogeriatric ward. The control group consisted of 44 cognitively healthy volunteers from the local community, including caregivers and relatives of patients, matched to the clinical group by age, sex, and educational level. All control participants were screened to exclude neurological, psychiatric, and significant systemic disorders. All individuals enrolled in the study were subjected to a comprehensive screening protocol to rule out comorbidities potentially impacting motor or cognitive performance, thereby ensuring the validity of outcome interpretation.

The mean age of individuals in the AD group was approximately 72 years (SD ≈ 6.5), with an age range between 60 and 85 years. Women represented more than half of the participants in the AD group. The mean duration of formal education among individuals with AD was 12 years (SD ≈ 3), corresponding to a secondary or vocational level of educational attainment. Among individuals in the control group, the mean age was approximately 70 years (SD ≈ 6), with a sex distribution comparable to that of the AD group and an average duration of formal education of 13 years (SD ≈ 3). No statistically significant differences in demographic characteristics were observed between the AD and individuals from control groups. Cognitive functioning in individuals with Alzheimer’s disease was assessed using the Mini-Mental State Examination (MMSE), with a mean score of approximately 21 points (SD ≈ 3.5), consistent with mild to moderate dementia severity. In cognitively healthy individuals from the control group, MMSE scores fell within the normal range, with a mean score of 28 points (SD ≈ 1.5).

All participants were confirmed to be right-handed according to the Edinburgh Handedness Inventory, had corrected-to-normal vision, and showed no clinically significant motor impairments unrelated to dementia. Informed consent was obtained from each individual or their legal representative, and the study was approved by the local institutional review board in accordance with the Declaration of Helsinki.

### Procedure

The diagnosis of Alzheimer’s disease in the clinical group was established according to internationally recognised criteria (McKhann et al., 2011) and ICD-10 guidelines (World Health Organization, 1992). Cognitive functions were assessed using the Mini-Mental State Examination (MMSE), a screening tool for global cognitive performance (Folstein et al., 1975). To evaluate executive and attentional functions, the Trail Making Test (TMT) parts A and B was administered (Reitan, 1958). Handedness was determined using the Edinburgh Handedness Inventory (Oldfield, 1971).

Graphomotor performance was recorded with a digitizing tablet and analyzed using custom-developed software, enabling the extraction of temporal, kinematic, and pressure-related parameters (Table 1). This approach builds on previous studies demonstrating the diagnostic utility of handwriting and drawing analysis in differentiating mild cognitive impairment and Alzheimer’s disease (Werner et al., 2006). Signal processing included Fourier-based decomposition to quantify tremor-related oscillations, in line with established methods of biomechanical signal analysis (Oppenheim and Schafer, 2010).

**Table 1:** Operational definitions of graphomotor variables.

Feature	Category	Operational definition
Execution time	Temporal	Total time required to complete the task
Path length	Spatial / kinematic	Total distance travelled by the pen tip during task execution
Mean velocity	Kinematic	Average speed of pen movement during task execution
Maximum instantaneous velocity	Kinematic	Highest momentary pen movement velocity recorded during the task
Mean instantaneous velocity	Kinematic	Average value of instantaneous velocity across the movement trajectory
SD of instantaneous velocity	Kinematic variability	Variability of instantaneous movement velocity during task execution
Mean pressure	Pressure-related	Average relative force applied by the pen to the tablet surface
Maximum pressure	Pressure-related	Highest relative pen pressure recorded during the task
SD of pressure	Pressure variability	Variability of relative pen pressure during task execution
THD-derived spectral irregularity index	Frequency-domain / signal stability	Operational index of oscillatory irregularity derived from FFT-based analysis of pressure, velocity, or trajectory-deviation signals

The table summarizes temporal, kinematic, pressure-related, and frequency-domain parameters extracted from the tablet-based graphomotor tasks.

The assessments were conducted individually for each participant in a quiet, sound-controlled laboratory environment under standardized conditions, minimizing the presence of distracting stimuli.

During the assessment, the participant was seated at a desk in front of a computer connected to a graphic tablet equipped with an electronic pen. Prior to the commencement of the assessment, each participant received detailed verbal instructions regarding the task to be performed and completed a brief training trial to become familiar with the equipment’s functionality. The training trial was not included in the data analysis. The tablet’s stylus resembled a conventional writing instrument (such as a pen) and did not provide haptic feedback beyond the natural resistance of the sheet of paper placed on the tablet’s surface, allowing for an ecologically valid simulation of handwriting and drawing.

The experimental protocol included a series of graphomotor tasks selected to allow, as far as possible, the assessment of various aspects of fine motor control, visuospatial processing, and executive functioning. The participants were instructed to complete simple drawing tasks, including the copying of geometric figures and free-form drawing without a template. These tasks were designed to assess visuoconstructive skills and the integration of motor planning with visual–spatial guidance. Participants were asked to provide a handwritten signature in their typical manner, a task selected for its ecological validity and its reliance on highly automatized yet complex motor sequences. Participants completed a tablet-based digital adaptation of the Trail Making Test, parts A and B, which involved connecting sequential numbers (part A) and alternating between numbers and letters (part B) displayed on the tablet screen. The task allows for the assessment of psychomotor efficiency, divided attention, and cognitive flexibility, while simultaneously enabling the acquisition of detailed biophysical data on movement.

**Figure 1:**
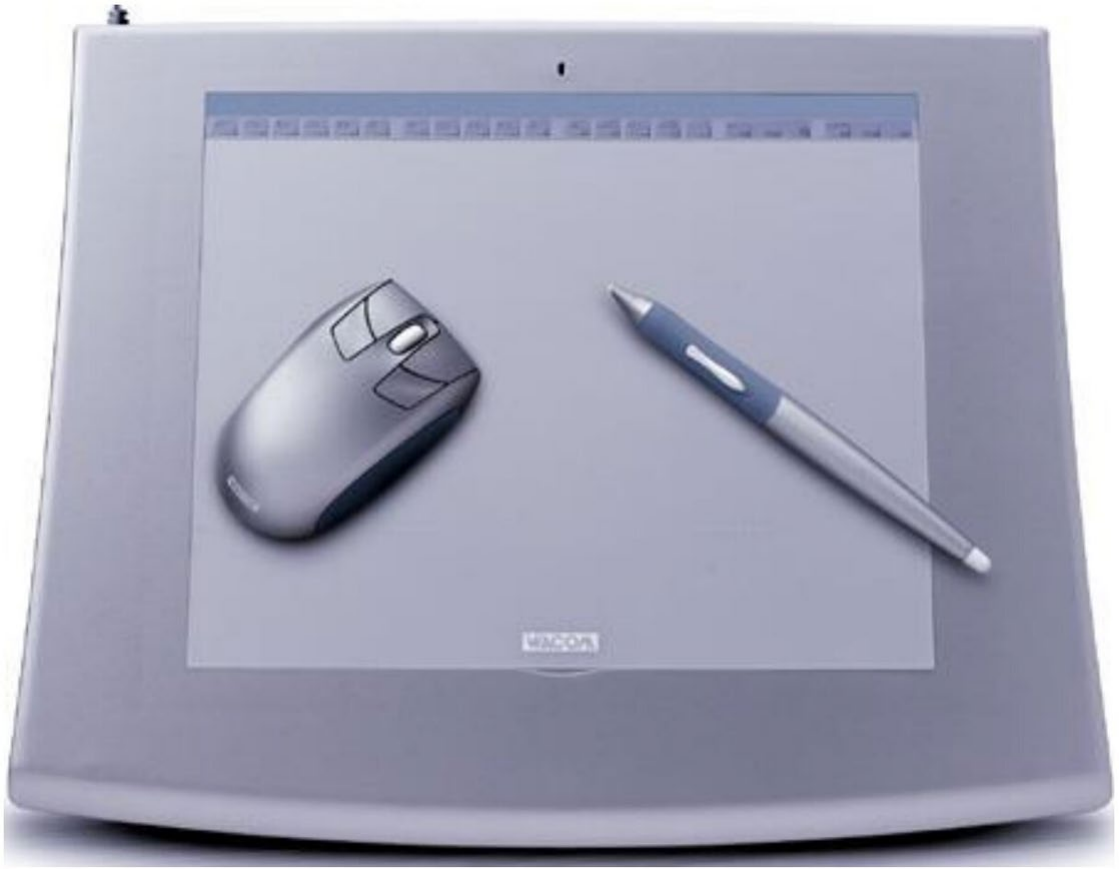
Tablet-based graphomotor assessment setup. The figure shows the Wacom Intuos2 digitizing tablet. The system captured pen-tip position, timing, movement velocity, relative pressure, and frequency-domain signal irregularity during signature, drawing, and Trail Making Test tasks.

Graphomotor data were collected using the original, author-developed MedTablet software, specifically designed for this type of research to enable quantitative assessment of psychomotor efficiency and visuospatial functioning. A Wacom Intuos2 tablet was used (A4 active area; 2,540 dpi resolution; 1,024 pressure levels; 200 Hz sampling rate) (Wacom Technology Corporation, 2003), connected to a computer (Figure 1). The software incorporated dedicated signal-processing algorithms, including fast Fourier transform (FFT) analysis, enabling the automatic extraction of temporal, kinematic, pressure-related, and tremor-characterization parameters (Raethjen and Deuschl, 2012; Elble and Koller, 1990). This author-developed platform enables precise and objective measurement of movement and assessment of motor performance, as well as motor-related cognitive functions, particularly those associated with working memory.

During task execution, the digitizing system continuously recorded the trajectory of the tablet pen tip both while in contact with the tablet surface and during in-air movements, providing high-resolution data on spatial coordinates, timing, velocity, and relative pen pressure. The recording enables the capture of subtle micromovements, such as tremor-related oscillations, which are subjected to quantitative and harmonic analysis (Fourier-based decomposition). The investigator was positioned in close proximity to the participant, monitoring correct task execution and providing clarification when necessary, while refraining from any direct assistance that could influence performance outcomes. Each participant completed the full task battery in a single session lasting approximately 30 to 40 min. The pace of task execution was self-regulated, with participants having the option to request short breaks as needed. All data were anonymized at the point of collection and stored securely for later analysis.

The digitizing system provided a rich set of raw data for each graphomotor task, including the pen-tip position in three-dimensional space (x: lateral, y: vertical, z: depth/height), temporal information with a precision of five milliseconds, and 1,024 dynamic relative pressure levels exerted by the tablet pen tip on the sheet of paper placed over the graphic tablet surface. Digital data capturing the position of the tablet pen during task execution enables detailed quantitative assessment of movement parameters. Temporal parameters were extracted to characterize the total duration of each individual task and to quantify the time spent in direct contact between the tablet pen tip and the sheet of paper placed on the digitizing surface, relative to the time during which the pen tip remained disengaged from the writing surface. Comparison of the time spent in direct contact between the tablet pen tip and the writing surface with the time spent out of contact enables the analysis of both active stroke execution and contact interruptions. These temporal distinctions serve as a basis for indirect inferences about cognitive functioning related to planning, hesitation, and corrective processes during task performance. The deconstruction of the motor trajectory of tablet pen movements enabled the calculation of average stroke duration and inter-stroke intervals, providing insight into task execution fluency. These temporal metrics may indirectly reflect cognitive processes such as planning efficiency, execution certainty, cognitive uncertainty, and decision-related conflicts.

Spatial measures were derived based on the length of the tablet pen trajectory and its variability within the designated drawing area, enabling assessment of the extent, stability, and precision of motor execution. The total path length of the tablet pen trajectory served as an indirect index of motor efficiency, while deviations from the expected movement pattern were interpreted as indicators of visuospatial control integrity, encompassing aspects of spatial planning, trajectory monitoring, and corrective regulation. Kinematic indicators were calculated based on temporal changes in the tablet pen tip position, allowing for the extraction of velocity and acceleration profiles. Mean and peak velocity values, along with intra-trial velocity variability, were analyzed to detect motor slowing, transient accelerations, and trajectory irregularities indicative of disrupted execution dynamics. Acceleration patterns were analyzed to assess the smoothness of transitions between successive movement phases. Sudden changes in acceleration values may indicate motor coordination impairments, difficulties in movement planning, or cognitive uncertainty, thereby allowing for an indirect evaluation of execution efficiency.

Continuous recording of relative pressure applied by the tablet pen tip during surface contact enabled the extraction of mean force levels and their variability (standard deviation), providing an indirect measure of motor execution stability. Relative pressure and its variability (standard deviation) provide an objective measure of motor signal stability and the ability to maintain consistent control over fine upper limb movements, serving as an indirect indicator of executive functioning and visuomotor coordination. Increased variability in relative pressure was interpreted as a marker of diminished motor precision and compromised neuromuscular stability, suggesting potential deficits in fine force modulation and execution control mechanisms.

Frequency-domain analyses were performed using Fourier transformation (FFT) of biomechanical velocity signals and relative pressure force to identify tremor-related oscillations. FFT analyses were performed on three derived one-dimensional time series: pen-pressure signal, instantaneous velocity signal, and trajectory-deviation signal relative to the template. Signals were sampled at 200 Hz, detrended, and analysed within the 1–16 Hz frequency range, corresponding to the tremor-related band used in the MedTablet processing pipeline. Because handwriting and drawing are non-stationary and non-periodic behaviours, the THD parameter was not interpreted as classical harmonic distortion of a periodic waveform. Instead, it was used as an operational spectral-irregularity index reflecting the relative contribution of higher-frequency oscillatory components to the total movement signal. THD-derived spectral irregularity index was calculated as a measure of signal irregularity and micro-movement noise, serving as an indirect indicator of neuromotor dysfunction. Graphomotor task performance was comprehensively characterized across the domains of execution time, spatial accuracy, kinematic fluency, motor force regulation, and signal stability. This multidimensional movement strategy was designed to enhance sensitivity to subtle motor and visuospatial deficits in Alzheimer’s disease and to enable rigorous comparison with control participants through the analysis of biomarker parameter values.

The experimental protocol consisted of three tablet-based graphomotor tasks designed to assess complementary aspects of psychomotor efficiency, visuomotor coordination, fine motor control, and executive functioning. All tasks were performed with a digital pen on test sheets placed over the active surface of the digitizing tablet, allowing continuous recording of pen movement, timing, trajectory, and relative pressure during task execution.

In the signature task, participants were instructed to sign their name with the digital pen in their habitual manner. The signature was to be legible and executed in the style most typical for the participant, either as the full first name and surname or as the surname alone, depending on the participant’s usual signing habit. This task was included because personal signature production represents a highly practiced and largely automatized graphomotor activity, with relatively low cognitive demand compared with the other tasks in the protocol. It therefore provided a measure of fine motor execution and motor fluency during an overlearned writing sequence.

**Figure 2:**
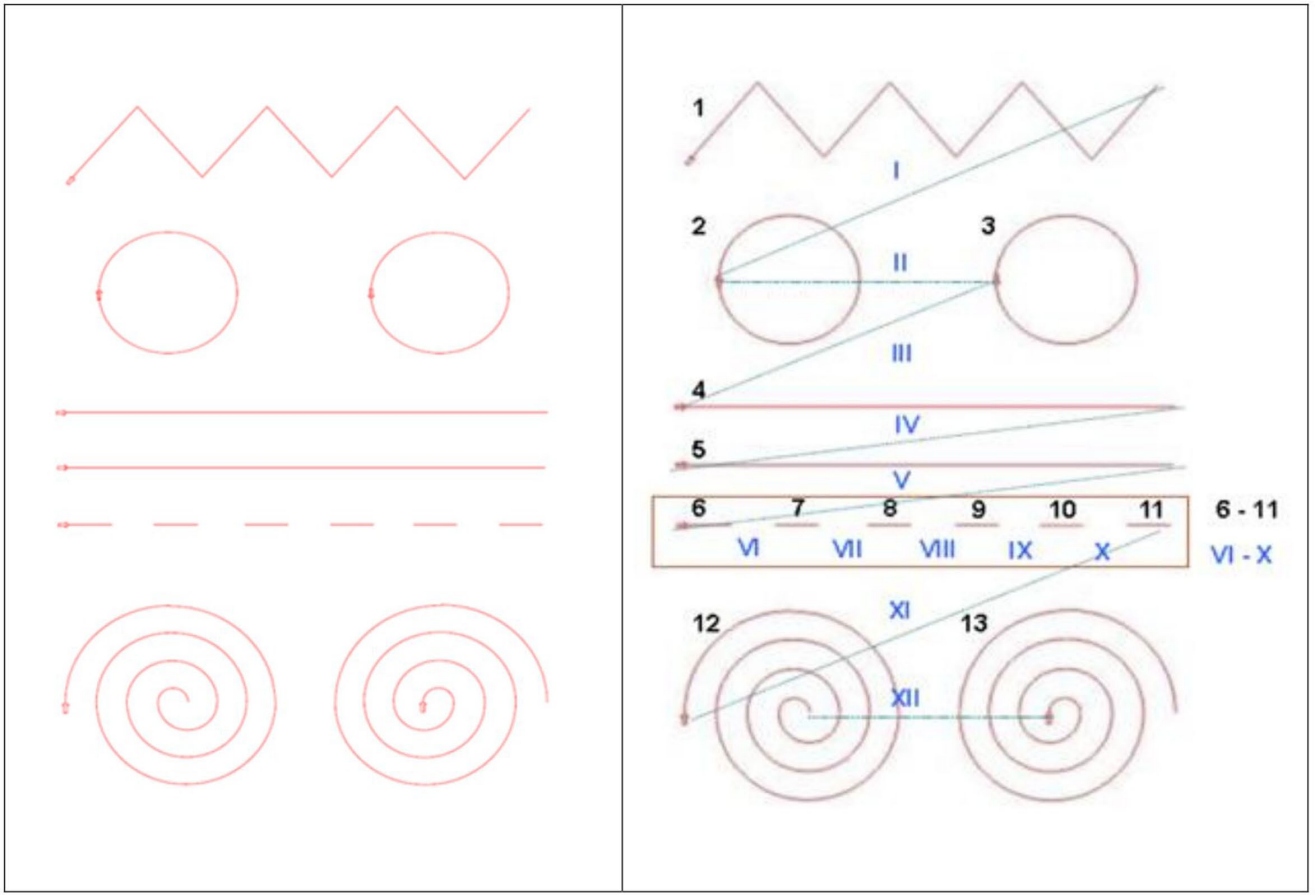
Standardized drawing sheet used in the graphomotor task (Vibro). The figure illustrates the set of 13 geometric shapes employed to assess visuoconstructive abilities, motor planning, and fine motor control. The task included reproduction of simple and complex forms (e.g., straight and dashed lines, zigzag, spiral, and polygonal figures), arranged in a fixed order to elicit increasing levels of motor and visuospatial demand. Shapes II-X targeted fluency and coordination under varying biomechanical load, while shapes XI-XII required integration of visuospatial planning with continuous motor execution. The standardized sheet served as the reference template for quantitative analysis of temporal, kinematic, and pressure-related parameters recorded with the digitizing tablet (Gorzelańczyk et al., 2008; Faron et al., 2013; Olzak et al., 2006).

In the drawing task, participants were instructed to reproduce a standardized set of thirteen graphic forms presented on the test sheet shown in Figure 2. The figure served as the standardized reference material for the graphomotor assessment. The set included simple and complex geometric forms, including straight lines, dashed lines, zigzag lines, spirals, circular forms, and polygonal figures. The selection, order, and drawing direction of these forms were designed to elicit different levels of visuoconstructive, visuomotor, and graphomotor demand. Short dashed lines required repeated initiation and termination of movement, allowing assessment of motor fluency and movement segmentation. Zigzag and spiral forms required continuous visuomotor guidance and varying degrees of upper-limb coordination, including changes in movement direction and elbow extension, thereby enabling assessment of coordination and movement precision under changing biomechanical conditions. The standardized drawing sheet served as the reference template for subsequent quantitative analysis of temporal, kinematic, pressure-related, and frequency-domain movement features.

The Trail Making Test parts A and B were administered in a tablet-based format. In part A, participants connected sequentially ordered numbers, whereas in part B they alternated between numbers and letters. These tasks were used to assess psychomotor speed, visuomotor coordination, divided attention, working memory, attentional shifting, and executive control (Reitan, 1958; Lezak et al., 2004). In contrast to the conventional paper-and-pencil administration, the tablet-based version enabled continuous recording of pen trajectories and movement dynamics during task execution. This allowed the classical completion-time measure to be supplemented with detailed biomechanical and biophysical parameters, including movement trajectory, velocity, and pressure-related features. Thus, the digital adaptation of the TMT provided additional information on motor execution and movement-control irregularities while preserving the core cognitive demands of the original task.

### Statistical analysis

Statistical analyses were carried out with the STATISTICA software package (StatSoft Inc., Tulsa, OK, United States). Descriptive statistics were computed for all variables prior to hypothesis testing, including indices of central tendency and dispersion such as the mean, standard deviation, median, interquartile range, and distributional shape. “Group homogeneity with respect to demographic variables was assessed using appropriate comparative tests. The normality of distributions for graphomotor parameters was evaluated with the Shapiro–Wilk test and by visual inspection of histograms and Q–Q plots. Homogeneity of variances was examined using Levene’s test.

Because most graphomotor variables showed non-normal distributions or heterogeneity of variance, all primary between-group comparisons were performed using the Mann–Whitney *U* test. Parametric tests were not used for primary inference and are therefore not reported. For qualitative variables, χ^2^ tests were applied to verify the equivalence of individuals across groups.

Receiver operating characteristic (ROC) curve analyses were performed for each parameter to assess the diagnostic utility of graphomotor features. The area under the ROC curve (AUC) was estimated with 95% confidence intervals as a measure of the test’s diagnostic power and the certainty of its ability to discriminate between individuals across groups. Thresholds for acceptable, good, and excellent diagnostic performance were established, with AUC values ≥0.70 regarded as indicating minimally acceptable classification accuracy. Sensitivity and specificity were assessed at optimal cut-off points, with statistical significance defined as *p* < 0.05. The analytic strategy was designed to avoid bias from violations of parametric assumptions and to ensure that the discriminative capacities of graphomotor parameters were assessed using rigorous and transparent statistical procedures.

## Results

### Signature task

Movement parameters recorded during the signature task were compared between patients with Alzheimer’s disease and healthy controls. Detailed results are presented in Table 2. The mean path length during signing in individuals with Alzheimer’s disease was not significantly different from that observed in the control group (AD: 27.78 cm ± 13.63; controls: 28.47 cm ± 13.39; *p* > 0.05). The signing time was nominally 48% longer in individuals with Alzheimer’s disease than in healthy controls (16.76 s vs. 11.30 s), (*p* = 0.047; q = 0.113); however, this effect did not survive FDR correction for multiple comparisons within the signature task family and should therefore be interpreted as an exploratory trend rather than a robust task-specific marker. In contrast, the velocity-related parameters remained statistically significant after FDR correction. Mean velocity, maximum instantaneous velocity, mean instantaneous velocity, and variability of instantaneous velocity showed robust group differences, indicating that kinematic slowing and instability, rather than execution time alone, constitute the most reliable signature-task abnormalities in Alzheimer’s disease. In individuals with Alzheimer’s disease, the mean stroke velocity was 21% lower (2.36 cm/s ± 1.97 vs. 2.99 cm/s ± 1.64; *p* = 0.0017), the maximum instantaneous velocity was nearly 30% lower (14.38 cm/s ± 6.00 vs. 20.42 cm/s ± 12.77; *p* < 0.001), and the mean instantaneous velocity was 28% lower (2.74 cm/s ± 1.46 vs. 3.82 cm/s ± 1.74, *p* = 0.0002) compared with the corresponding values in the control group. Signature execution time was significant only before correction and should be interpreted cautiously. After FDR correction within task-specific families, the most robust findings were pressure variability in the drawing task, velocity-related parameters in the signature task, and temporal, kinematic, and pressure-related abnormalities in TMT-A and TMT-B. Borderline nominal findings, including signature execution time, were not retained as robust effects after correction. The standard deviation of mean instantaneous velocity was significantly higher in patients with Alzheimer’s disease compared with healthy controls, indicating greater intra-individual variability and motor instability during the signing process in AD. The mean motor parameters and movement variability suggest that psychomotor efficiency is globally lower in individuals with Alzheimer’s disease relative to healthy controls.

**Table 2:** Graphomotor parameters obtained during the signature task.

Parameter	AD mean	AD SD	Control mean	Control SD	*p*-value nominal	FDR *q*
Path length (cm)	27.78	13.63	28.47	13.39	>0.05	
Execution time (s)	16.76	18.92	11.3	5.85	0.047112	0.113069
Mean velocity (cm/s)	2.36	1.97	2.99	1.64	0.001729	0.005187
Max inst. Velocity (cm/s)	14.38	6	20.42	12.77	0.000208	0.001248
Mean inst. Velocity (cm/s)	2.74	1.46	3.82	1.74	0.000389	0.001556
SD inst. Velocity (cm/s)	2.29	0.96	3.21	1.43	0.000109	0.001248
Mean pressure	754.21	139.56	765.73	129.55	>0.05	
Max pressure	970	103.12	983.59	80.31	>0.05	
SD pressure	165.95	30.58	159.95	33.54	>0.05	
THD pressure	1.35	0.7	1.5	0.8	>0.05	
THD trajectory	0.51	0.26	0.56	0.29	>0.05	
THD inst. Velocity	1.53	0.87	1.64	0.8	>0.05	

Temporal, kinematic, pressure-related, and frequency-domain parameters recorded during the signature task in patients with Alzheimer’s disease and cognitively healthy controls. Data are presented as mean and standard deviation (SD), together with the corresponding statistical comparisons between groups. *Nominal p value* indicates the statistical significance before correction for multiple comparisons, whereas the *FDR q* value represents the corresponding probability value after adjustment using the Benjamini–Hochberg false discovery rate procedure.

No statistically significant differences were observed in the relative pen pressure exerted on the tablet surface between individuals with Alzheimer’s disease and healthy controls. The mean relative pressure was slightly lower in Alzheimer’s patients (754.21 ± 139.56) compared with controls (765.73 ± 129.55; *p* > 0.05), and the mean maximal relative pressure values were also nonsignificantly lower in the Alzheimer’s group (970.00 ± 103.12 vs. 983.59 ± 80.31). The mean variability of the mean relative pen-tip pressure on the tablet surface, assessed as the standard deviation, was nonsignificantly higher in patients with Alzheimer’s disease compared with healthy controls (165.95 ± 30.58 vs. 159.95 ± 33.54). The findings indicate that pressure control during signing is relatively preserved in individuals with Alzheimer’s disease; however, the velocity-related parameters point to reduced psychomotor efficiency in these patients compared with healthy controls. Spectral analysis of graphomotor signal oscillations, expressed as THD-derived spectral irregularity index coefficients, revealed consistently lower values across all evaluated parameters in individuals with Alzheimer’s disease compared with controls, although these differences did not reach statistical significance. THD-derived spectral irregularity index values for pressure oscillations, trajectory deviations, and mean instantaneous velocity were slightly lower in AD patients than in controls, but none of these differences were statistically significant (*p* > 0.05). Mean values of movement parameters in individuals with Alzheimer’s disease were compared with those of the control group using the Mann–Whitney U test. Statistically significant differences were found for mean execution time, mean velocity, mean maximum instantaneous velocity, mean instantaneous velocity, and mean variability of instantaneous velocity (all *p* < 0.05). No statistically significant differences were observed for mean path length, mean relative pen-tip pressure on the tablet surface, or mean values of harmonic distortion indices.

### Drawing task

Movement parameters recorded during the drawing task were compared between patients with Alzheimer’s disease and healthy controls. Detailed results are presented in Table 3. No statistically significant differences were found in the values of mean drawing length and mean drawing time between individuals with Alzheimer’s disease and those in the control group. The mean drawing length in individuals with Alzheimer’s disease (AD) was 186.36 cm (±35.82), while in the control group it was 183.60 cm (±11.31). No statistically significant difference in mean drawing time was observed between individuals with AD and the individuals from control group (148.47 s ± 61.05 vs. 143.89 s ± 55.63; *p* > 0.05). The distributions of kinematic parameter values in individuals with Alzheimer’s disease largely overlapped with those of the individuals from control group. The mean drawing velocity in patients with Alzheimer’s disease (AD) was 1.44 cm/s (±0.55), which was nearly identical to that observed in the individuals from control group (1.43 cm/s ± 0.46). The maximum mean instantaneous velocity was slightly lower in patients with Alzheimer’s disease (14.83 cm/s ± 13.69) compared to controls (16.75 cm/s ± 11.90), whereas the mean instantaneous velocity was marginally higher in the AD group (1.39 cm/s ± 0.54) than in controls (1.27 cm/s ± 0.38). No statistically significant differences (*p* > 0.05) were found in the mean kinematic values of individuals with Alzheimer’s disease compared to healthy controls.

**Table 3:** Results of the drawing task.

Parameter	AD mean	AD SD	Control mean	Control SD	*p*-value
Drawing length (cm)	186.36	35.82	183.6	11.31	>0.05
Execution time (s)	148.47	61.05	143.89	55.63	>0.05
Mean velocity (cm/s)	1.44	0.55	1.43	0.46	>0.05
Max instantaneous velocity (cm/s)	14.83	13.69	16.75	11.9	>0.05
Mean instantaneous velocity (cm/s)	1.39	0.54	1.27	0.38	>0.05
SD instantaneous velocity (cm/s)	1.07	0.32	0.99	0.25	>0.05
Mean pressure	810.37	141.66	852.62	132.02	>0.05
SD pressure	174.12	29.38	151.65	23.4	*p* < 0.00005
Max pressure	994.36	66.93	1001.93	65.09	>0.05
THD pressure oscillations	0.63	0.34	0.63	0.19	>0.05
THD trajectory deviations	0.59	0.11	0.62	0.13	>0.05
THD instantaneous velocity	1	0.36	0.97	0.21	>0.05

Comparison of temporal, spatial, kinematic, pressure-related, and frequency-domain graphomotor parameters obtained during the drawing task in patients with Alzheimer’s disease and cognitively healthy controls. Data are presented as mean and standard deviation (SD) together with the corresponding statistical comparisons between groups.

The mean relative pressure force in patients with Alzheimer’s disease differed significantly from that of healthy controls, demonstrating discriminative potential. The mean relative pressure exerted by individuals with Alzheimer’s disease was lower (810.37 ± 141.66) than in the individuals in the control group (852.62 ± 132.02), while the mean relative maximum pressure was also slightly lower (994.36 ± 66.93 vs. 1001.93 ± 65.09). Notably, however, the standard deviation of mean relative pressure (SD_PRES) was significantly higher in individuals with Alzheimer’s disease (174.12 ± 29.38) compared to controls (151.65 ± 23.40; *p* = 0.000025), which may indicate greater irregularity and variability of applied pressure during the drawing task. These results suggest that, despite comparable mean values of relative pressure and relative maximum pressure, patients with Alzheimer’s disease exhibit greater instability in fine motor control, as reflected in fluctuations of pen pressure on the tablet surface during the drawing task. The Mann–Whitney *U* test analysis confirmed that, among all examined value drawing parameters, only the standard deviation of mean pressure (MN_SD_PRES) significantly distinguished patients with Alzheimer’s disease from controls (*Z* = 4.13; *p* = 0.000037), with an area under the ROC curve (AUC) of 0.729 (95% CI: 0.639–0.819). Variability of pen pressure appears to be a sensitive biomarker in the drawing task, whereas other temporal, kinematic, and spectral indices lack diagnostic value.

### Trail making test a and B

The mean completion time, kinematic parameters, and relative pen-pressure values obtained during Trail Making Test part A (TMT-A) were compared between individuals with Alzheimer’s disease and healthy controls (Table 4). Patients with AD required substantially more time to complete the task, with mean execution time being approximately 84% longer than in the control group. In addition, the total drawing path length was significantly greater in individuals with AD, indicating reduced efficiency and less optimal movement organization during task performance. Patients with Alzheimer’s disease also demonstrated significantly impaired movement dynamics. Mean movement velocity during TMT-A was more than 30% lower than in healthy controls, while maximum instantaneous velocity was reduced by nearly 20%. Variability of instantaneous velocity was likewise significantly lower in the AD group, suggesting diminished flexibility and adaptability of motor execution. Overall, these findings indicate generalized psychomotor slowing and reduced capacity to generate and maintain rapid movements in individuals with Alzheimer’s disease. Pressure-related parameters further differentiated the groups. During TMT-A performance, individuals with AD exhibited significantly lower mean relative pen pressure together with markedly greater pressure variability compared with healthy controls. Although no significant difference was observed for maximum pen pressure, the increased variability of pressure in the AD group may reflect impaired force regulation and weakened fine motor control during drawing movements. Analysis of THD-derived spectral irregularity index revealed significantly greater distortion of drawing trajectories in patients with Alzheimer’s disease, indicating reduced movement precision and greater deviation from the intended path. In contrast, THD measures related to velocity and pen pressure did not differ significantly between groups. Statistically significant differences were observed across temporal, kinematic, and pressure-related parameters during TMT-A performance (*p* < 0.05). Non-parametric analyses confirmed that the digitized version of the test is sensitive to disturbances in psychomotor efficiency, movement accuracy, and pressure regulation associated with Alzheimer’s disease.

**Table 4:** Trail making test A and B graphomotor parameters.

Parameter	AD mean	AD SD	Control mean	Control SD	*p*-value
TMT A - Drawing length (cm)	548.58	153.9	449.5	79.38	*p* = 0.0002
TMT A - Execution time (s)	114.47	50.66	62.12	19.73	*p* < 0.00001
TMT A - Mean velocity (cm/s)	5.18	1.95	7.57	1.74	*p* < 0.00001
TMT A - Max inst. Velocity (cm/s)	35.38	13.56	43.72	11.47	*p* = 0.004
TMT A - SD inst. Velocity (cm/s)	5.46	1.75	6.84	1.9	*p* = 0.001
TMT A - Mean pressure	812.61	141.08	889.5	137.41	*p* = 0.02
TMT A - SD pressure	187.22	32.11	165.71	26.72	*p* = 0.0006
TMT A - THD trajectory	0.77	0.3	0.67	0.23	*p* = 0.03
TMT B - Mean velocity (cm/s)	3.46	1.81	5.39	1.95	*p* < 0.00001
TMT B - Max inst. Velocity (cm/s)	19.53	9.21	27.56	9.71	*p* < 0.00001
TMT B - Mean inst. Velocity (cm/s)	3.09	1.57	3.09	1.04	>0.05
TMT B - SD inst. Velocity (cm/s)	4.04	1.79	5.03	1.56	*p* = 0.005
TMT B - Mean pressure	765.81	144.67	849.38	130.02	*p* = 0.003
TMT B - SD pressure	198.15	40.23	152.4	29.71	*p* < 0.00001
TMT B - THD pressure	1.04	0.46	1.21	0.55	*p* = 0.04

Temporal, spatial, kinematic, pressure-related, and frequency-domain parameters recorded during the tablet-based Trail Making Test parts A and B in patients with Alzheimer’s disease and cognitively healthy controls. Data are presented as mean and standard deviation (SD) together with between-group statistical comparisons. TMT-B completion status was additionally considered because some participants were unable to complete the task.

Task completion was analysed as an additional categorical outcome. Failure to complete TMT-B was substantially more frequent in the AD group than in controls, indicating that inability to complete the task may itself represent a clinically meaningful marker of executive and psychomotor impairment. However, because incomplete performance restricts quantitative analysis of trajectory-derived parameters, TMT-B kinematic results were interpreted with caution. Performance on TMT part B by individuals with Alzheimer’s disease is more impaired compared with performance on TMT-A, with the majority of patients (85%) found to be unable to complete the task. A full analysis of drawing time and path length in individuals with Alzheimer’s disease performing the test is limited. Among those with Alzheimer’s disease who attempted the TMT-B, significant differences were observed compared with healthy controls. In individuals with Alzheimer’s disease performing the TMT-B, mean velocity was more than 35% lower and mean maximum instantaneous velocity was 41% lower compared with healthy controls, both differences were highly statistically significant. The mean instantaneous velocity did not differ significantly between individuals with Alzheimer’s disease and healthy controls. However, the variability of instantaneous velocity was significantly reduced in the Alzheimer’s group, which may indicate limited motor adaptability (reflecting reduced psychomotor efficiency compared with healthy individuals). It was found that the mean maximum pen pressure on the tablet surface in individuals with Alzheimer’s disease did not differ significantly from that of healthy controls. However, patients with Alzheimer’s disease demonstrated a significantly lower mean pen pressure and nearly 50% greater pressure variability compared with the mean pen pressure of healthy individuals. It was found that the mean THD value of pressure oscillations was significantly lower in individuals with Alzheimer’s disease compared with healthy controls. In contrast, the standard deviation and the mean THD values related to velocity did not differ significantly between the groups. Non-parametric comparisons of mean velocity, maximum velocity, variability of instantaneous velocity, mean pen pressure, and pressure variability during TMT-B performance in individuals with Alzheimer’s disease and healthy controls revealed statistically significant differences (*p* < 0.05 for all parameters).

### Receiver operating characteristic (ROC) analysis

**Figure 3:**
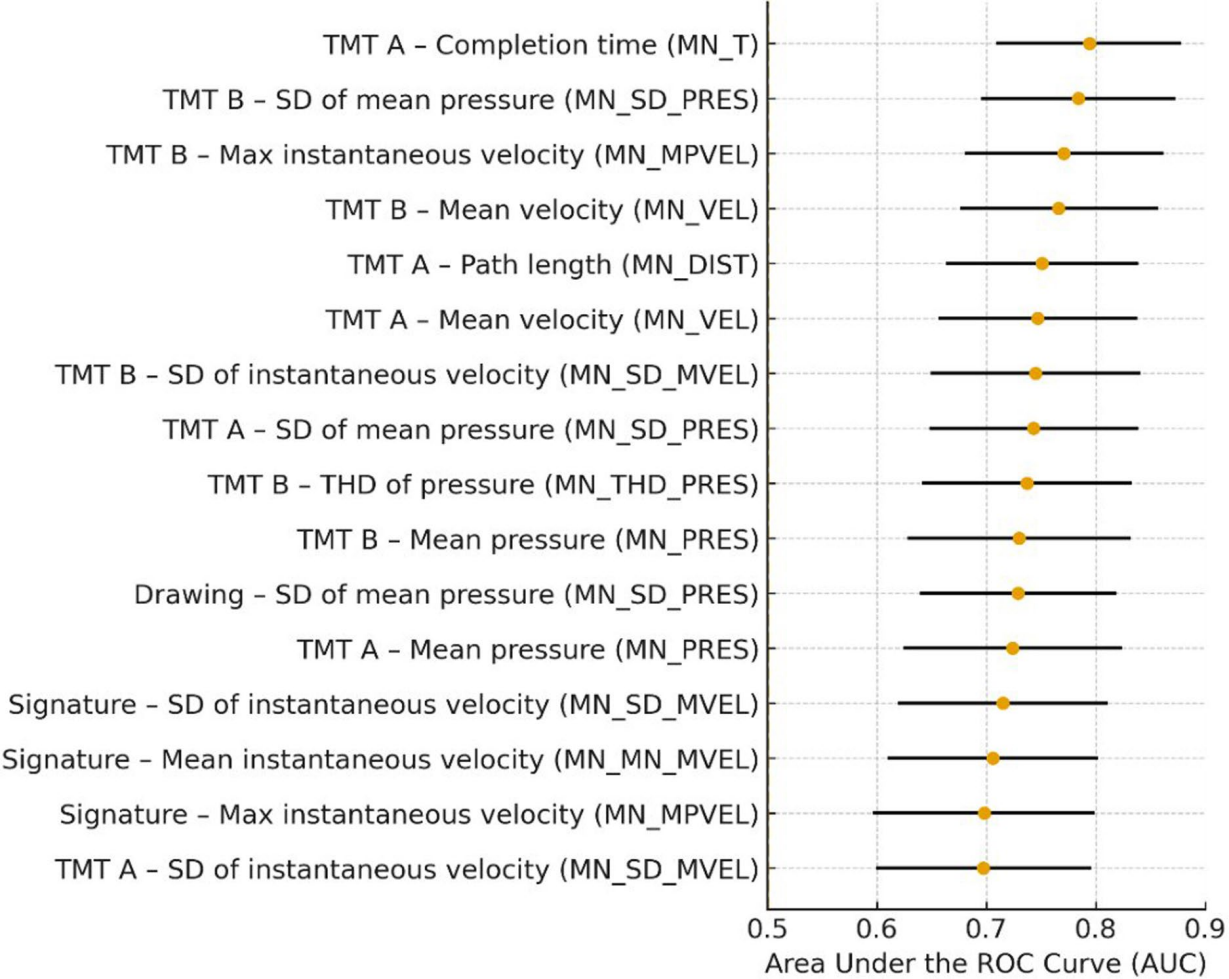
Forest plot of ROC performance for graphomotor parameters. Points represent AUC values with 95% confidence intervals; the dashed line indicates chance-level discrimination.

Receiver operating characteristic (ROC) curves were constructed to assess the diagnostic performance of the extracted graphomotor measures for all parameters that demonstrated statistically significant differences between the individuals from groups under comparison. The analysis revealed that several indices achieved AUC values falling within the range generally considered acceptable for clinical discrimination (Table 5). Among the evaluated parameters, the highest diagnostic effectiveness was observed for the total completion time in part A of the Trail Making Test, with an AUC value of 0.79. The obtained results indicate that the longer completion time of a relatively simple sequencing task in individuals with Alzheimer’s disease, compared with healthy controls, constitutes a marker with high sensitivity and specificity for Alzheimer’s disease. The diagnostic effectiveness of the TMT-A completion time parameter is determined by the clear separation between groups, with minimal overlap in distributions, allowing for balanced sensitivity and specificity at the optimal cut-off (Figure 3). Likewise, in the cognitively more demanding Trail Making Test part B, the maximum pen-velocity parameter achieved an AUC of 0.77. This finding highlights that, in addition to the longer test completion time observed in individuals with Alzheimer’s disease compared with healthy controls, there is also a limitation in generating and sustaining high peak movement velocities, which reflects reduced psychomotor efficiency and impaired executive motor control in Alzheimer’s disease. The ROC curve analysis for test completion time confirmed strong discriminatory power, particularly in identifying patients with moderate deficits who were able to complete the task but exhibited abnormal kinematic parameters compared with healthy controls.

**Table 5:** AUC values with 95% confidence intervals, and the corresponding sensitivity and specificity parameters are presented for the measures meeting the criteria of diagnostic significance.

Test	Variable	AUC	CI_low	CI_high	Sensitivity	Specificity
Drawing	MN_SD_PRES	0.729	0.639	0.819	0.66	0.76
Signature	MN_MPVEL	0.698	0.596	0.799	0.86	0.79
Signature	MN_MN_MVEL	0.706	0.61	0.802	0.84	0.65
Signature	MN_SD_MVEL	0.715	0.619	0.811	0.87	0.8
TMT A	MN_DIST	0.751	0.663	0.839	0.89	0.8
TMT A	MN_T	0.794	0.709	0.878	0.85	0.8
TMT A	MN_VEL	0.747	0.656	0.838	0.77	0.89
TMT A	MN_SD_MVEL	0.697	0.599	0.796	0.85	0.82
TMT A	MN_PRES	0.724	0.624	0.824	0.68	0.74
TMT A	MN_SD_PRES	0.743	0.648	0.839	0.81	0.76
TMT B	MN_VEL	0.766	0.676	0.857	0.69	0.82
TMT B	MN_MPVEL	0.771	0.68	0.862	0.89	0.67
TMT B	MN_SD_MVEL	0.745	0.649	0.841	0.78	0.82
TMT B	MN_PRES	0.73	0.628	0.832	0.75	0.82
TMT B	MN_SD_PRES	0.784	0.695	0.873	0.72	0.7
TMT B	MN_THD_PRES	0.737	0.641	0.833	0.84	0.68

AUC, area under the receiver operating characteristic curve; CI, confidence interval.

The standard deviation of tablet-pen pressure applied to the surface was identified as a parameter of notable diagnostic significance, achieving an AUC of 0.78 across tasks. The variability of the mean tablet-pen pressure on the surface indicates instability and inconsistency in the applied force among individuals with Alzheimer’s disease compared with healthy controls, reflecting impaired fine motor control and reduced psychomotor efficiency. The diagnostic relevance of variability in tablet-pen pressure applied to the surface is of particular importance, since this parameter supplements time- and velocity-related indices by capturing a distinct motor domain that is less affected by practice effects, thus offering potential utility for longitudinal monitoring.

The conducted ROC analyses confirmed that graphomotor parameter values encompassing temporal, kinematic, and pressure-related domains allow differentiation between individuals with Alzheimer’s disease and healthy controls with accuracy ranging from moderate to high. It is worth emphasizing that the comprehensive nature of the analyzed parameters indicates that applying a multivariate approach, integrating time, velocity, and pressure variability, may enhance classification accuracy compared with single measures. The presented findings confirm that digital graphomotor parameter analysis provides potential non-invasive biomarkers with clear translational potential for subclinical diagnostics, early staging, and clinical evaluation of individuals with Alzheimer’s disease.

## Discussion

Previous studies have shown that digital handwriting, drawing, and clock-drawing paradigms can capture subtle cognitive and motor impairment in MCI and Alzheimer’s disease. More recent approaches have expanded this framework by combining drawing trajectories with additional behavioural modalities, including hand motion, facial expression, and other sensor-derived features. Compared with these multimodal systems, the present study focuses on a narrower but clinically simpler and technically reproducible pen-tablet protocol. Its contribution is not the introduction of graphomotor analysis as a completely new concept, but the systematic comparison of several task contexts: signature, drawing, and digital TMT A/B, within one AD case–control sample, with simultaneous assessment of temporal, kinematic, pressure-related, and harmonic parameters. This design allows us to examine which graphomotor domains are task-specific and which appear across different levels of cognitive-motor demand.

Psychomotor functions constitute a key domain for the integration of cognitive and emotional processes (Giustino et al., 2023; Pessoa, 2013). The analysis of movement dynamics makes it possible to capture subtle changes that often precede clinically recognizable symptoms of neurological and psychiatric disorders (Gorzelańczyk, 2011; Olzak et al., 2006; Walther and Morrens, 2015). We applied digitized graphomotor tasks on a tablet-based digital pen recording, including a signature test, figure copying, and the Trail Making Test (TMT) (Reitan, 1958). The signature test evaluates procedural memory, fine motor control, and motor automatization (van Drempt et al., 2011). The figure copying test assesses executive functions, visuospatial perception, and visuomotor coordination (Blaskewitz et al., 2009; Kourtis et al., 2019; Shin et al., 2006). The Trail Making Test (TMT) Part A assesses processing speed, selective attention, psychomotor efficiency, and visuomotor coordination, whereas Part B additionally evaluates cognitive flexibility, set-shifting, working memory, error monitoring, and the inhibition of automatic responses (Bowie and Harvey, 2006; Lezak et al., 2012; Reitan, 1958; Sánchez-Cubillo et al., 2009). By using a graphic tablet during task performance, data on the values of biophysical movement parameters were collected (Drotár et al., 2014; Garre-Olmo et al., 2017; Planton et al., 2017).

Quantitative approaches, using digital tools such as graphic tablets and biomechanical signal acquisition systems, now play an increasingly important role in the precise measurement of movement parameters (Drotár et al., 2014; Garre-Olmo et al., 2017; Simonetti et al., 2024). Such approaches allow the study of phenomena beyond conventional clinical assessment, supporting subclinical diagnostics of psychomotor functions (Mittal and Walker, 2007; Perez et al., 2021). Experimental studies indicate that biomechanical signal analysis can differentiate healthy individuals from patients with neurodegenerative, psychiatric, or addictive disorders (Drotár et al., 2014; Lasoń et al., 2010; Walecki et al., 2013). Graphomotor tasks, including signature, figure copying, and the Trail Making Test using an electromagnetic digitizing tablet, allow precise recording of parameters such as reaction time, movement trajectory length, pressure, and tremor spectrum. Analysis in the time, frequency, and nonlinear domains reveals distinctive motor disturbance patterns that can act as diagnostic and prognostic markers (Llinàs-Reglà et al., 2017). Such an approach is especially relevant in Alzheimer’s disease, where conventional neuropsychological assessments largely emphasize cognitive deficits (Tsoi et al., 2015). Growing evidence suggests that early neuropsychiatric changes affect motor–cognitive integration and movement fluency (Haaland et al., 2017; Leisman et al., 2016; Mittal and Walker, 2007; Walther and Strik, 2012).

With its sensitivity and objectivity, graphomotor analysis can complement conventional diagnostics, offering insight into cortico-subcortical loop function and their progressive breakdown in neurodegenerative disease (Drotár et al., 2014; Ferrer-Ramos et al., 2025; Garre-Olmo et al., 2017). Neuroimaging studies show that writing and drawing engage writing-specific regions as well as networks shared with other graphomotor tasks (Seitz and Roland, 1992). Writing and drawing activate complex cortico-subcortical networks supporting motor planning, control, and visuospatial integration (Katanoda et al., 2001; Planton et al., 2013; Planton et al., 2017; Seitz and Roland, 1992) Alzheimer’s disease involves both cortical dysfunction and progressive disruption of cortico-subcortical loops integrating cognitive, emotional, and motor functions, as reflected in our findings (Seoane et al., 2024). Early motor and fluency impairments in AD reflect behavioral correlates of cortico-subcortical loop dysfunction (Garre-Olmo et al., 2017; Halliday, 2017; Mega and Cummings, 1994; Zhang et al., 2025). Cortico-striato-thalamic pathways, linking prefrontal and limbic regions with the basal ganglia and thalamus, are central to the regulation of complex behaviors (Alexander et al., 1986; Halliday, 2017; Lajoie et al., 2017; Mega and Cummings, 1994; Zhang et al., 2025). Progressive disruption of these circuits may underlie the motor slowing, reduced velocity, and greater pressure variability observed in our cohort during graphomotor tasks relative to controls (Alexander and Crutcher, 1990; Katanoda et al., 2001; Mega and Cummings, 1994; Planton et al., 2013, 2017; Zhang et al., 2025).

Graphomotor movement analysis can reveal parameters with strong potential as high-sensitivity biomarkers (Cavedoni et al., 2020; Garre-Olmo et al., 2017). The longer task completion times, reduced movement speed, and greater variability of pressure observed in individuals with AD compared with healthy controls may reflect the disintegration of cortico-striato-thalamo-cortical circuits, which physiologically support fluent execution of movement sequences, visuospatial integration, and executive control (Haber and Knutson, 2010). AD pathology affects not only cortical structures but also their dynamic interactions with the basal ganglia and thalamic relay systems, resulting in impaired temporal coordination and movement precision. Neuropathological evidence of fronto-parietal and temporo-hippocampal disconnection likely underlies irregularities in graphomotor task performance (Lee et al., 2020; Zhang et al., 2025). Cortico-subcortical loops underpin automated but flexible motor routines, including handwriting and the Trail Making Test (Alexander et al., 1986; Mega and Cummings, 1994; Seoane et al., 2024; Garre-Olmo et al., 2017).

Our findings demonstrate differences in graphomotor performance between individuals with Alzheimer’s disease and healthy controls. Patients with AD required significantly more time to complete the tasks and exhibited clear reductions in psychomotor efficiency, reflected by lower mean and peak pen-movement velocities (*p* < 0.05). In the signature task, this slowing was evident throughout the entire writing sequence rather than being restricted to isolated strokes, suggesting a generalized disruption of movement fluency and automatized motor execution. Individuals with AD also displayed less stable movement dynamics, characterized by irregular velocity profiles, greater heterogeneity of stroke length, and increased variability in trajectory curvature (Drotár et al., 2014; Garre-Olmo et al., 2017). In addition, pen-tip pressure regulation was more inconsistent, as indicated by higher standard deviation values relative to controls. Together, these abnormalities point to impaired neuromuscular coordination and diminished fine motor control during precision-dependent handwriting tasks (Cavedoni et al., 2020; Garre-Olmo et al., 2017). ROC analyses further confirmed the diagnostic relevance of these measures, with temporal and kinematic parameters achieving AUC values between 0.71 and 0.77, corresponding to moderate-to-good discriminatory accuracy (Cavedoni et al., 2020; Garre-Olmo et al., 2017; Hanley and McNeil, 1982). Our study should be interpreted within the rapidly growing field of digital graphomotor biomarkers for neurodegenerative disorders. While several studies have already demonstrated the diagnostic value of digital handwriting and drawing analysis (Garre-Olmo et al., 2017; Yamada et al., 2022; Fan et al., 2025), the present study extends this evidence by systematically comparing signature, drawing, and digital TMT A/B within the same Alzheimer’s disease cohort using a unified acquisition and signal-processing framework.

Crucial markers are not only the longer task completion times but also the greater variability of drawing speed in patients with AD compared with healthy controls. The combination of these two indicators, time and variability, enhances the clinical value of the analysis and improves diagnostic accuracy in differentiating individuals with AD from healthy subjects. In patients with Alzheimer’s disease, signatures are produced more slowly and less fluently, with greater variability in movement and pen-tip pressure compared with healthy individuals. From a clinical perspective, the execution of a well-known, highly automated motor program, namely the personal signature, repeatedly performed throughout life, is impaired in Alzheimer’s disease and meets the criteria for bradykinesia, while also pointing to a broader spectrum of neurodegenerative pathology involving the control of fine movements and their integration with cognitive planning (Roalf et al., 2018). During the signature task, pen-tip pressure was a less sensitive marker than maximum and mean instantaneous velocity or velocity variability. AUC values of 0.698–0.715 support the role of these parameters as potential digital biomarkers for early detection and monitoring of motor dysfunction in AD (Cavedoni et al., 2020; Vrahatis et al., 2023). Importantly, correction for multiple comparisons modified the interpretation of some task-specific findings. In the signature task, execution time showed only nominal significance and did not survive FDR adjustment. Therefore, prolonged signing time should not be regarded as a robust independent marker in this dataset. By contrast, velocity-related parameters remained significant after correction, supporting the interpretation that kinematic slowing and reduced movement fluency are more stable graphomotor alterations in Alzheimer’s disease than total execution time during an overlearned task such as signing.

Disruptions in cortico-subcortical loop function in Alzheimer’s disease help explain why even well-learned actions, such as producing a personal signature, are performed more slowly and with greater variability compared with healthy individuals (Garre-Olmo et al., 2017). Greater intra-individual variability in movement parameters and pen-tip pressure in individuals with AD, compared with healthy controls, may serve as a behavioral marker of reduced stability within cortico-subcortical loops, reflecting impaired control and difficulties in adapting movements to task demands (Wriessnegger et al., 2020). Motor impairments in AD may emerge alongside, or even before, cognitive decline, in line with theoretical models and clinical observations (Seoane et al., 2024). The figure-copying task in requires a lower degree of learning and repetition compared with the signature task. It was found that drawing time and movement path length did not differ significantly between individuals with Alzheimer’s disease and healthy controls. In AD, mean, maximum, and instantaneous drawing speeds and their variability were comparable to those of controls.

A markedly greater variability of pen-tip pressure on the tablet surface was observed in individuals with Alzheimer’s disease compared with healthy controls, indicating impaired stability of fine motor regulation. ROC analyses further demonstrated that pressure-related parameters effectively discriminated between AD and control groups, supporting their potential utility as biomarkers for the early detection of motor dysfunction within the clinical spectrum of Alzheimer’s disease. In the digitized version of the Trail Making Test (TMT), both parts A and B revealed statistically significant group differences across temporal, kinematic, and pressure-related measures. As expected, individuals with Alzheimer’s disease required significantly more time to complete both TMT-A and TMT-B and also showed prolonged intervals when connecting successive points (*p* < 0.001). Performance on TMT-B was particularly impaired, reflecting the additional executive demands associated with alternating between numbers and letters. Compared with healthy controls, patients with AD demonstrated lower mean and peak pen-movement velocities, indicating reduced psychomotor fluency. Their movement trajectories were also less continuous and more irregular, with greater instability of velocity profiles, suggesting impaired motor coordination and reduced execution stability. Furthermore, individuals with AD exhibited weaker regulation of pen-tip pressure, characterized by lower average pressure values and increased variability during task execution. These disturbances were consistently present across both TMT conditions, supporting the presence of combined psychomotor and executive dysfunction in Alzheimer’s disease.

Individuals with Alzheimer’s disease more frequently produced elongated movement trajectories and showed greater deviations from the optimal path connecting successive points than healthy controls. These abnormalities were especially evident during TMT-B, suggesting impaired visuospatial planning and reduced integration of sequential motor actions. Task execution in the AD group was also characterized by more frequent interruptions and prolonged pen-up periods, which may reflect deficits in planning and executive control. ROC curve analyses confirmed the diagnostic relevance of these graphomotor parameters. In TMT-A, longer completion time, reduced maximum velocity, and increased variability of relative pen pressure achieved AUC values ranging from 0.74 to 0.79, indicating good discriminative performance. For TMT-B, AUC values ranged between 0.72 and 0.77, with combined temporal and kinematic indices providing the most balanced sensitivity and specificity. These findings demonstrate that clinically meaningful information is derived not only from overall completion time, but also from detailed movement dynamics recorded during task execution. Performance in both TMT-A and TMT-B revealed generalized psychomotor slowing in individuals with Alzheimer’s disease, accompanied by reduced movement velocity, impaired pressure regulation, and increased instability of motor execution. The presence of these abnormalities across both test conditions suggests that the observed graphomotor deficits are linked to combined psychomotor and executive dysfunction. Digital graphomotor analysis therefore appears capable of detecting subtle cortico-subcortical disturbances that may serve as early indicators of disease progression. High-resolution motor biomarkers can complement traditional neuropsychological tests, improving diagnostic accuracy (Cavedoni et al., 2020; Drotár et al., 2014; Vrahatis et al., 2023).

The present results suggest that digitized graphomotor assessment may represent a sensitive approach for detecting motor and cognitive impairments associated with Alzheimer’s disease (AD). Across the signature task, figure-copying task, and Trail Making Test (TMT), individuals with AD demonstrated abnormalities in both temporal and kinematic domains, including prolonged completion times, reduced movement velocity, and increased intra-individual variability relative to healthy controls.

These observations are consistent with previous studies indicating that graphomotor disturbances in AD and mild cognitive impairment (MCI) reflect broader deficits in visuospatial processing, executive functioning, and fine motor control. Signature performance in AD was characterized less stable pen movement, while pressure regulation remained relatively preserved. This pattern suggests that even highly automatized motor routines become progressively disrupted during the course of the disease, supporting earlier findings that learned handwriting loses fluency as neurodegeneration advances. In the figure-copying task, the observed abnormalities were less pronounced; however, variability of pen pressure emerged as a particularly sensitive indicator of early motor instability and impaired neuromuscular regulation. Performance on the TMT reflected the combined influence of psychomotor slowing, impaired pressure control, and executive dysfunction. Individuals with Alzheimer’s disease showed marked impairment in TMT-A, whereas the majority were unable to complete TMT-B, emphasizing the high vulnerability of cognitive flexibility and set-shifting processes to disease-related decline. ROC curve analyses further demonstrated that graphomotor parameters achieved acceptable-to-good diagnostic accuracy. Temporal and velocity-related measures provided the strongest discriminatory performance, although pressure variability contributed additional diagnostic information. Consistent with previous reports, multimodal digital biomarkers combining several movement domains appeared to outperform single-parameter metrics. These findings support the growing body of evidence highlighting the diagnostic and prognostic potential of digital pen- and tablet-based assessments, particularly in the context of early detection and monitoring of Alzheimer’s disease pathology.

The implementation of digital biomarkers raises both ethical and practical considerations. Evidence suggests that disclosing biomarker information to individuals without dementia may have complex psychological consequences and could influence the clinical picture, underscoring the need for carefully designed communication protocols (van der Schaar et al., 2023). At the same time, the rapid development of digital technologies in Alzheimer’s disease offers new opportunities for monitoring and early diagnosis, as demonstrated by large-scale research analyses and international collaborative initiatives (Lott et al., 2024). These developments should also be interpreted within the broader context of dementia prevention, intervention, and care strategies highlighted by the Lancet Commission, which emphasize the integration of innovative tools into existing public health frameworks (Livingston et al., 2020).

The present findings should be interpreted as exploratory evidence supporting the potential utility of tablet-derived graphomotor parameters as candidate digital biomarkers. They do not establish clinical validation. Independent replication, external validation, multivariable modeling, and longitudinal assessment are required before these measures can be considered validated diagnostic biomarkers.

## Limitations

Several limitations should be acknowledged. First, the cross-sectional case–control design precludes conclusions about the longitudinal predictive value of graphomotor measures. Future studies should evaluate whether these markers predict conversion from MCI to AD and track progression across disease stages. Second, although our sample size was moderate, external validation in larger and more diverse cohorts is required to establish generalizability. Third, the majority of participants were recruited from a memory clinic, which may introduce selection bias toward clinically typical AD phenotypes. Fourth, the tablet-based setup requires standardization of hardware and software to ensure reproducibility across sites, as differences in sampling rate, surface friction, or pen sensitivity could influence measurement precision. Fifth, a specific limitation concerns TMT-B. Because most patients with AD were unable to complete this task, analyses of TMT-B kinematic and pressure variables were restricted to a subgroup of patients who attempted or completed sufficient portions of the task. This subgroup is likely to represent individuals with less severe executive impairment, which may introduce selection bias and limit generalizability of TMT-B-derived estimates. For this reason, TMT-B results should be interpreted primarily as exploratory and as evidence of task feasibility and disease-related impairment rather than as definitive diagnostic thresholds. Finally, while we attempted to control for demographic factors such as age and education, subtle effects of motor comorbidities or hand dominance cannot be fully excluded.

Although FDR correction was applied to reduce the risk of false-positive findings, the number of analysed graphomotor variables remains large relative to the sample size. The results should therefore be interpreted as exploratory and hypothesis-generating. This is particularly relevant for borderline nominal effects, such as signature execution time, which did not remain significant after correction for multiple comparisons.

## Future directions

Future research should focus on integrating graphomotor parameters with other modalities of digital biomarkers to create complex, multimodal diagnostic profiles. Incorporating temporal, kinematic, and pressure-based indices into broader datasets, encompassing signals from mobile devices, smartwatches, fitness bands, smart glasses, and sensors embedded in clothing or jewelry that continuously monitor physiological and motor parameters, may enhance sensitivity and specificity in detecting early neurodegenerative changes (Dagum, 2018). Particularly promising is the application of artificial intelligence and machine learning methods to extract latent patterns from complex motor signals, which may enable the identification of novel, non-obvious predictive features (Winchester et al., 2023).

Another important direction is the expansion of biomarker research to extra-neuronal structures, such as the retina, where pathology may serve as a non-invasive indicator of neurodegenerative processes in Alzheimer’s disease (Alber et al., 2024). In parallel, increasing attention is being directed toward oculomotor assessments, including analyses of saccades, fixations, and smooth pursuit, which show significant alterations in patients with mild cognitive impairment and Alzheimer’s disease. These parameters may represent sensitive indicators of early cognitive and visuospatial dysfunction, and their potential as digital biomarkers has been confirmed in systematic reviews and meta-analyses (Opwonya et al., 2022). Combining ophthalmic, oculomotor, and graphomotor-cognitive digital biomarkers could yield a new generation of diagnostic tools characterized by high resolution and the ability to monitor changes over time.

Future studies should develop multivariable prediction models integrating temporal, kinematic, pressure-related, and task-completion variables. These models should be evaluated using internal cross-validation and then tested in independent external cohorts. Such an approach would allow assessment of whether combined graphomotor signatures outperform single-parameter ROC analyses.

A key challenge for clinical translation remains the development of large, normative datasets that allow algorithms to be calibrated against demographic and cultural variability. In parallel, standardized and easily deployable platforms must be developed to enable the use of graphomotor and oculomotor tests in outpatient and home settings. Only such an approach will ensure scalability and real-world applicability in routine diagnostics and disease monitoring.

## Conclusion

Digitized graphomotor analysis detects subtle but consistent alterations in fine motor execution, visuospatial planning, and executive functioning in Alzheimer’s disease. The present findings highlight temporal slowing, velocity reduction, and pressure variability as key domains of dysfunction, and demonstrated acceptable diagnostic accuracy across tasks. These results support the use of graphomotor parameters as objective, scalable, and non-invasive digital biomarkers that can complement conventional neuropsychological testing. With further validation, such tools may enable earlier detection, more precise disease monitoring, and improved clinical trial stratification in Alzheimer’s disease.

## Data Availability

The datasets generated and/or analyzed during the current study are not publicly available because they contain sensitive clinical data and public sharing was not covered by the participants’ informed consent or the ethics approval. De-identified data may be made available by the corresponding author upon reasonable request, subject to approval by the appropriate ethics committee and applicable data protection regulations.
